# Evaluation of the reliability of lower extremity alignment measurements using EOS imaging system while standing in an even weight-bearing posture

**DOI:** 10.1038/s41598-021-01646-z

**Published:** 2021-11-11

**Authors:** Byung Woo Cho, Tae-Ho Lee, Sungjun Kim, Chong-Hyuk Choi, Min Jung, Koo Yeon Lee, Sung-Hwan Kim

**Affiliations:** 1 Department of Orthopedic Surgery, Gangnam Severance Hospital, Yonsei University College of Medicine, 211 Eonju-ro, Gangnam-gu, Seoul, Republic of Korea; 2 Arthroscopy and Joint Research Institute, Yonsei University College of Medicine, Seoul, Republic of Korea; 3 Department of Radiology, Gangnam Severance Hospital, Yonsei University School of Medicine, Seoul, Republic of Korea; 4 Department of Orthopedic Surgery, Severance Hospital, Yonsei University College of Medicine, Seoul, Republic of Korea; 5 Center for Clinical Imaging Data Science Center, Research Institute of Radiological Science, Yonsei University College of Medicine, Seoul, Republic of Korea

**Keywords:** Musculoskeletal system, Bone imaging, Radiography, Whole body imaging

## Abstract

This study aimed to analyze the reproducibility and reliability of the alignment parameters measured using the EOS image system in both limbs while standing with an even weight-bearing posture. Overall, 104 lower extremities in 52 patients were analyzed retrospectively. The patients stood with an even load over both lower extremities then rotated 15° in both directions. Two EOS images were acquired and 104 pairs of lower extremities were compared according to the position of the indexed lower extremities. Then, the inter-observer reliability of the EOS system and the inter-modality reliability between EOS and computed tomography (CT) were evaluated. Femoro-tibial rotation (FTR) and tibial torsion demonstrated a significant difference between the anterior and posterior positions of the indexed lower extremity. In the inter-observer reliability analysis, all values except for FTR and tibial torsion demonstrated good or very good reliability. In the anterior position, FTR demonstrated moderate, and tibial torsion demonstrated poor reliability. In the posterior position, both FTR and tibial torsion demonstrated poor reliability. In the reliability analysis between the three-dimensional (3D) EOS model and 3D CT images, all measurements of the femur demonstrated very good reliability, but measurements of the tibia did not. For the coronal and sagittal alignment parameters measured by the EOS 3D system with rotated standing posture, except for the measurement including tibial torsion., there were no significant difference for either position of the indexed extremities with high agreement between the observers as well as with the CT 3D model.

## Introduction

Accurate assessment of lower extremity alignment is imperative for preoperative and follow-up patient evaluations in orthopedic practices, such as corrective osteotomies and joint replacement surgeries^1–3^. Evaluations of femoral and tibial torsion as well as coronal and sagittal lower extremity alignments are essential for orthopedic surgeons in diagnosing and treating patients of all ages with lower extremity diseases^4^. Coronal and sagittal alignments, most commonly used in actual clinical practice, are evaluated using full-length weight-bearing anteroposterior radiographs of the lower extremities, which is a conventional method using two-dimensional (2D) images. However, it has been reported that measuring the alignment of the lower extremities with 2D images can be affected by knee flexion and knee joint rotation in the axial plane^5^. It has also been reported that flexion contracture in the sagittal plane has a negative effect on the evaluation of the coronal alignment of the lower extremities^6^. Since most osteoarthritic knees are associated with flexion contracture and rotational deformation, the effect of these deformities must be considered in the evaluation of the coronal alignment of the lower extremities^7,8^.

To overcome this issue, several three-dimensional (3D) methods, such as computed tomography (CT), magnetic resonance imaging (MRI), and intraoperative navigation systems^9^, can be utilized to increase the accuracy of the assessments of lower extremity alignment^10–12^. However, these methods do not reflect the actual weight-bearing status; therefore, the evaluations may have potential errors and are accompanied by concerns regarding their cost-effectiveness^13–17^. In conventional 2D CT, the reliability and reproducibility of the alignment assessments are limited by positional variables that affect the determination of the anatomical axis and the measurement accuracy^4^. Additionally, this method includes concerns about radiation exposure^18^. In this regard, the EOS Imaging System (EOS Imaging Inc., Paris, France) is a good alternative as it simultaneously provides both weight-bearing full-length lower extremity images and a reference point for the alignment measurements in the three-dimensional (3D) space. The EOS imaging system simultaneously acquires two vertical plane images through a single scan and generates a 3D model of the lower extremity using semi-manual fitting of individualized shapes over the bones using a dedicated software (sterEOS, France). Additionally, the assessments of the clinical alignment of the lower extremity, including femoral and tibial torsion, are automatically calculated in the 3D coordinated system^19^. The EOS imaging system not only provides 3D alignment measurements of the lower extremity in a weight-bearing state but also uses a lower radiation dose compared with CT^18,20,21^. Therefore, it has the advantage of more accurately reflecting the actual physiological state of lower extremity alignment than the preexisting modality.

**Figure 1:**
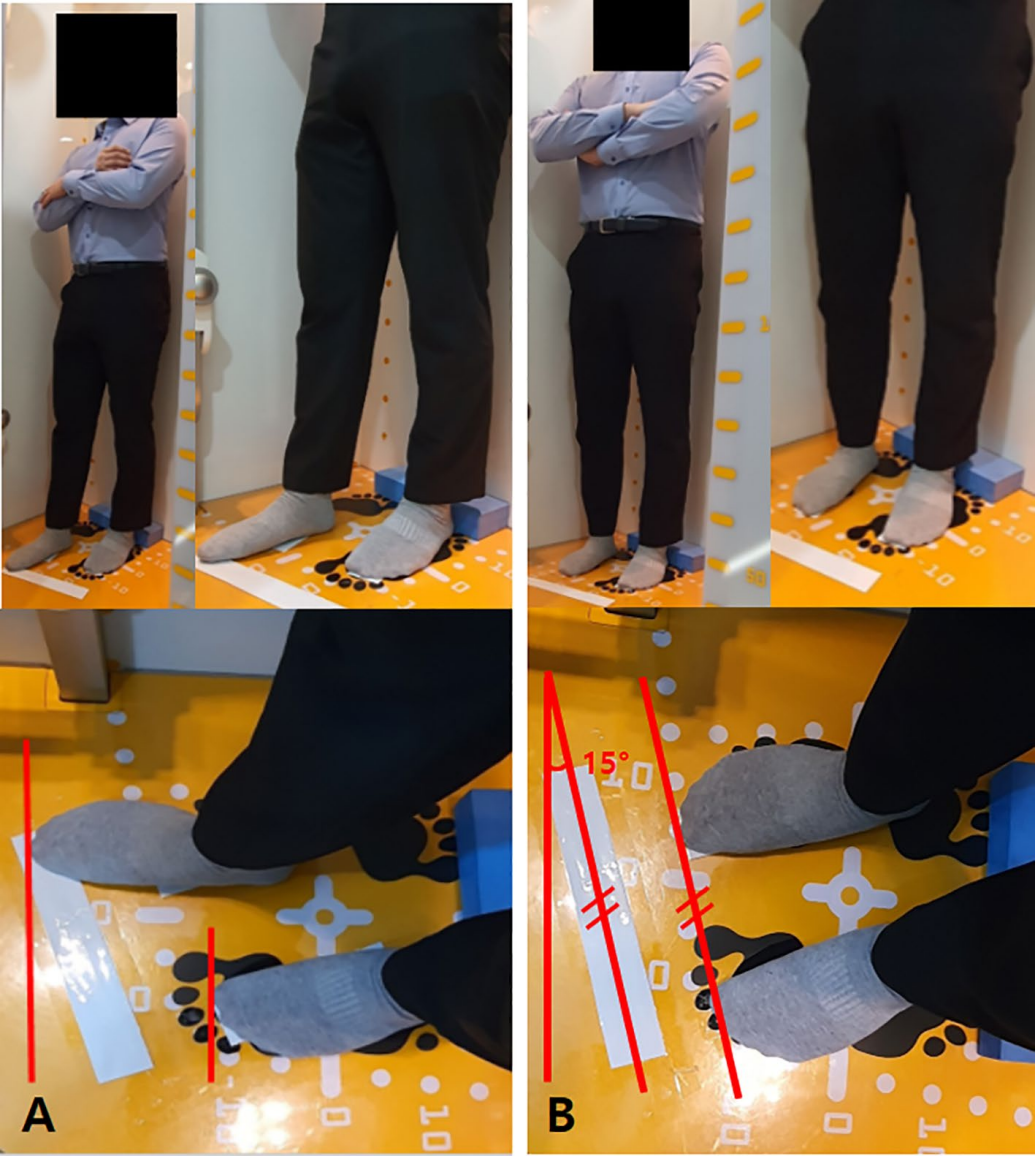
(**A**) Conventional standing posture for the EOS imaging system. (**B**) Modified rotated posture with even load distribution on both lower extremities. The red lines indicate the position and orientation of both feet.

However, in the sagittal view of the EOS images, a fundamental problem is the difficulty in identifying each anatomical and mechanical reference point when both lower extremities are superimposed. A commonly suggested method to avoid such superimpositions is to slightly spread the feet slightly forward and back such that the lower extremities do not overlap in the sagittal images (Fig. 1-A)^22^. However, unlike the standard standing posture, this posture does not transmit an even load over both extremities; additionally, there is a concern that the inter-segmental alignment of the femur and tibia may vary^23–25^. One of the methods to solve these problems is obtaining biplanar X-ray images by rotating approximately 15° from the standard standing posture (Fig. 1-B). This method has the advantage that both lower extremities may not superimpose in the sagittal view while evenly loading them. However, one concern is that it may cause potential errors in semi-manual model adjustment, such as identification of the anatomical reference points. To the best of our knowledge, there have been no analyses of measurements using the EOS imaging system in such a rotated standing posture with an even distribution of load on the lower extremities^4,26,27^. Therefore, this study aimed to analyze the reproducibility and reliability of the alignment parameters of the lower extremities measured using the EOS image system while standing erect with an even weight-bearing posture. We hypothesized that the physiologic alignments of the lower extremities obtained through a rotated standard standing posture would show adequate reproducibility and reliability.

## Materials and methods

### Patients

Between December 2019 and February 2020, of 64 outpatients with osteoarthritis who were at least 19 years of age and underwent EOS imaging, the data of 104 lower extremities from 52 patients (mean age: 52.52 ± 13.92 years; 18 men and 34 women) were analyzed retrospectively. The exclusion criteria were as follows: (1) patients under 19 years of age; (2) patients with severe lower extremity deformities (such as following trauma or fracture), (3) patients who previously underwent knee joint replacement surgery, and (4) patients with prosthetic implants around the knee joint. Full-length lower extremity CT was performed simultaneously in 30/52 patients for comparative analysis with their respective EOS 3D models.

### Radiographic assessments and measurement protocol

**Figure 2:**
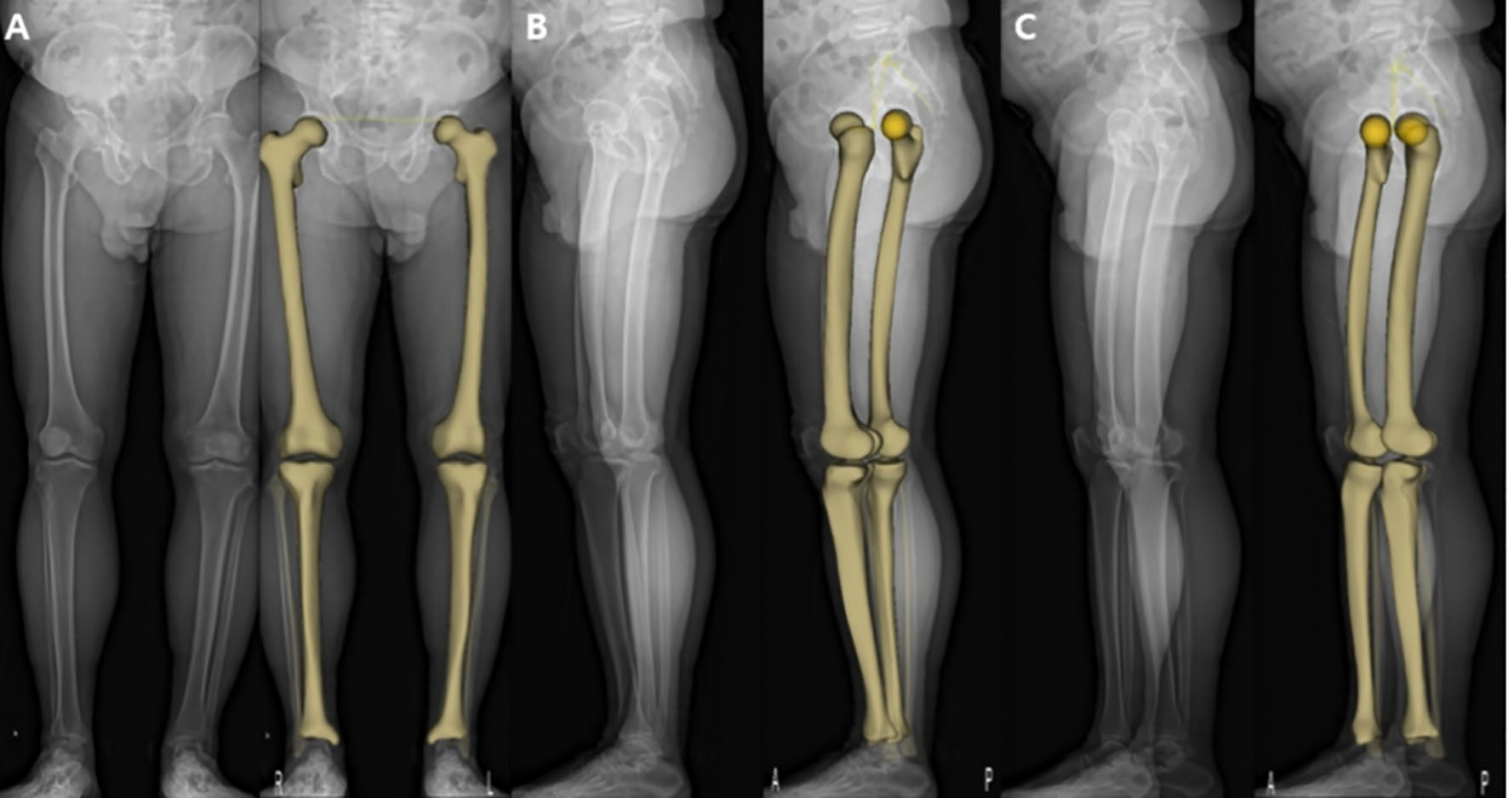
Three-dimensional (3D) model based on the EOS imaging system. Anteroposterior (**A**) and lateral radiographs (**B** and **C**) are acquired for 3D modeling. The 3D model is adapted to the osseous contours on radiographs.

**Figure 3:**
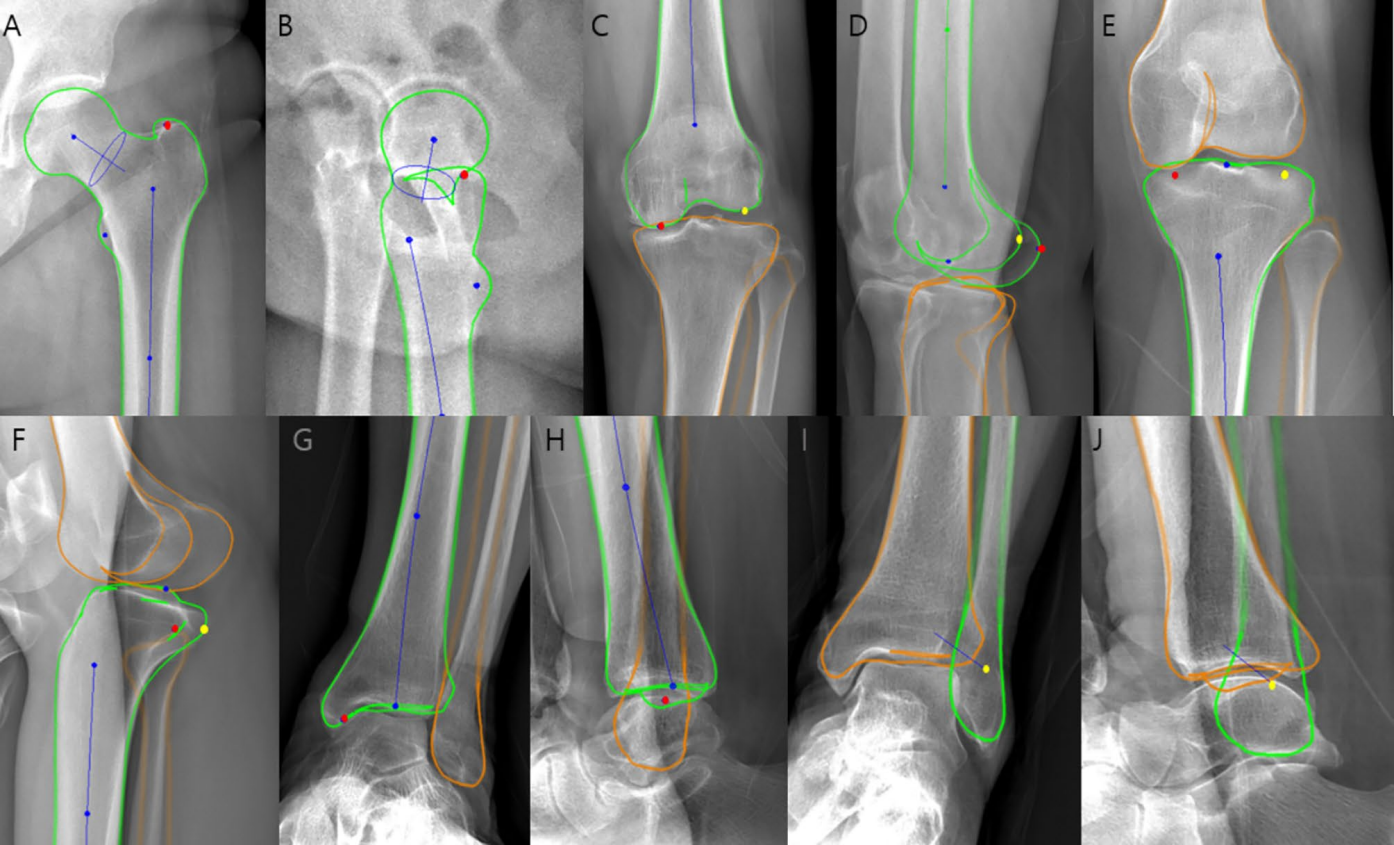
Three-dimensional model based on biplanar radiographs. (**A–J**) Computer model (green) is adapted semi-automatically to the osseous contours of the femur and tibia on anteroposterior (**A**, **C**, **E**, **G**, **I**) and lateral (**B**, **D**, **F**, **H**, **J**) radiographs. Three-dimensional models are built based on the fitting process, which is facilitated with standardized points on the osseous contour that can easily be adjusted by dragging and dropping. The key points that are crucial for torsion measurements are the greater trochanter at the hip (red point on **A**, **B**), the posterior contours of the femoral condyles (red points on **C**, **D**: medial condyle; yellow points on **C**, **D**: lateral condyle), the posterior contours of the medial and lateral aspects of the tibial head (red points on **E**, **F**: medial aspect; yellow points on E, F: lateral aspect), and the malleoli of the ankle joint (red points on G, H: medial malleoli; yellow points on I, J: lateral malleoli).

The anteroposterior and lateral images (tube voltage: 80 kV; tube current, 200 mA) were acquired simultaneously using the EOS imaging system with the patient in an upright posture to bear the physiological load. The patients applied the load evenly to both lower extremities with the patella facing forward and then rotated 15° in both directions to avoid superimposition of the anatomical references on lateral radiographs for a total of two EOS images (Fig. 1-B). Each 3D model was reconstructed from these biplanar X-ray images using a sterEOS workstation (EOS Imaging Inc., Paris, France) (Fig. 2). The 3D models of the lower extremities were obtained using semi-automatic adjustments of the anatomical reference points over the bone contours followed by fine manual manipulation (Fig. 3). The following variables were measured and analyzed: mechanical femorotibial angle (mFTA), flexion–extension angle (FEA), femorotibial rotation (FTR), hip-knee-shaft angle (HKSA), lateral distal femoral angle (LDFA), medial proximal tibial angle (MPTA), femoral torsion, and tibial torsion. The 104 pairs of lower extremities were divided into two groups (anterior position, n = 104; posterior position, n = 104) according to the position of the indexed lower extremity on the EOS image. An index extremity refers to the symptomatic side. Additionally, to measure the inter-observer reliability of the EOS system, the images were evaluated by two orthopedic surgeons blinded to patient information at 6-week intervals using the sterEOS software.

Inter-modality reliability between the EOS system and CT was evaluated for intra-segmental alignment. A CT scanner (GE Discovery CT 750 HD, GE Medical Systems) was used with the patient in the supine position with the hip joint extended and the thigh horizontal and parallel. Axial images of the hip, knee, and ankle (tube voltage, 120 kV; tube current, 185 mAs/slice; pitch factor, 0.426; matrix, 512 × 512; reconstructive thickness, 0.625 mm) were acquired without manipulation. The obtained CT Digital Imaging and Communications in Medicine file was transferred to a 3D reconstruction workstation (Mimics Research 21.0) to obtain a segmented 3D model as an STL file. An independent orthopedic surgeon trained to use the 3D reconstruction workstation created a full-length lower extremity 3D image by reconstructing 3D models of the femur and tibia. Subsequently, the lower limb alignment parameters were measured in reference to the axis, plane, and alignment values used on the EOS system.

### Definitions of lower limbs alignment parameters

The reference planes were defined as follows.

1. Frontal (coronal) femoral plane: Plane defined by the center of the femoral head and transepicondylar line (axis connecting the center of the condyles).
2. Sagittal femoral plane: Plane orthogonal to the frontal femoral plane.

The reference axes were defined as follows.

1. Femoral mechanical axis: Line connecting the center of the femoral head and the center of the trochlea.
2. Femoral anatomical axis: Line drawn down the center of the femur’s diaphysis.
3. Tibial mechanical axis (= tibial anatomical axis): Line connecting the center of the tibial spines and the center of the distal articular surface of the tibia.

Lower extremity alignment values were measured as follows. Intra-segmental alignments in 3D CT model were measured in the same way as the sterEOS software^22,28^.

1. Inter-segment alignment.

1. mFTA: On the frontal femoral plane, it represents the angle between the femoral mechanical axis and the tibial mechanical axis with a positive angle for valgus and a negative angle for varus alignments.
2. FEA: On the sagittal femoral plane, it represents the angle between the femoral mechanical axis and the tibial mechanical axis with a positive angle for flexion and a negative angle for extension.
3. FTR: It is the angle between the posterior bicondylar axis and the axis in contact with the posterior part of the tibial plateau with a positive angle for external rotation of the tibial plateau relative to the femoral condyles and a negative angle when it is internally rotated.
4. Intra-segment alignment:
5. LDFA: On the frontal femoral plane, it represents the acute angle between the femoral mechanical axis and the axis across the most distal point of the medial and lateral condyles and is always expressed as a positive angle.
6. MPTA: On the frontal femoral plane, this is the acute angle between the tibial mechanical axis and the line connecting the centers of the medial and lateral tibia plateaus.
7. HKSA: It represents the angle between the femoral mechanical axis and the femoral anatomical axis.
8. Femoral torsion: This represents the angle between the femoral neck axis and the bicondylar axis (axis between the centers of the spheres fitted to the medidal and lateral femoral condyles). It is measured by projecting it onto a plane perpendicular to the mechanical axis of the femur. A negative value indicates femoral retroversion.
9. Tibial torsion: This represents the angle between the line tangential to the posterior portion of the tibial plateau and the bimalleolar axis and is measured by projecting it onto a plane orthogonal to the mechanical axis of the tibia with a positive angle for external rotation (when the malleoli are turned externally in relation to the tibial plateau) and negative angle for internal rotation (when the malleoli are turned internally in relation to the tibial plateau).

### Statistical analysis

Paired *t*-tests and effect sizes were used to assess the differences in the measurements using the EOS imaging system according to the positions. Effect sizes were calculated for differences between the anterior and posterior positions of the lower extremity on the EOS 3D images using the standardized mean difference (mean difference divided by standard deviation) with 0.2, 0.5, and 0.8 considered as small, medium, and large effect sizes, respectively^29^. Single measurement intraclass correlation coefficients (ICCs) using two-way mixed effects were used to check for absolute agreement. ICCs were used to assess the inter-observer reliability of the values ​​measured by the two examiners using the EOS 3D system. ICC and Bland–Altman plots were used to assess the inter-method reliability between the CT 3D images and EOS system results^30,31^. The mean difference in the Bland–Altman plot was calculated to evaluate the bias of paired measurements, and the 95% limits of agreement (LOA) were calculated to evaluate the degree of agreement of paired measurements. According to the Altman guidelines, reliability of 0.0–0.20 = poor, 0.21–0.40 = fair, 0.41–0.60 = moderate, 0.61–0.80 = good, and 0.81–1.00 = very good^32^. Statistical analysis was performed using SPSS (version 25.0, IBM Inc., Armonk, NY, USA), and the statistical significance level was set at *P* < 0.05.

### Ethical approval

This study was approved by the Gangnam Severance Hospital Institutional Review Board (IRB No. 3-2020-0517) and the requirement for informed consent from the patients was waived because of the retrospective nature of the study. All methods were performed in accordance with the guidelines and regulations of Gangnam Severance Hospital IRB.

## Results

The lower limb parameters according to the position of the lower extremities (anterior and posterior) on the EOS 3D images are summarized in Table 1. Of the alignment values on the EOS images, FTR (95% confidence interval [CI], 2.18–4.32; effect size, 0.59) and tibial torsion (95% CI, 1.80–3.73; effect size, 0.55) demonstrated a statistically significant difference between the anterior and posterior positions. HKSA (95% CI, -0.75 to -0.13; effect size, -0.27) demonstrated a small effect size between the anterior and posterior positions. There were no statistically significant differences between the other alignment values.

**Table 1:** Comparison of the alignment values on the EOS 3D model according to the posture of the indexed lower extremity.

	Mean		Difference (95% CI)	Effect size	*P*-value
	Anterior	Posterior			
mFTA (°)	− 2.64 ± 3.83	− 2.54 ± 3.70	− 0.10 (− 0.34–0.14)	− 0.08	0.391
FEA (°)	2.49 ± 7.75	2.69 ± 7.85	− 0.2 (− 0.69–0.29)	− 0.08	0.426
FTR (°)	2.81 ± 5.28	− 0.44 ± 4.31	3.26 (2.18–4.32)	0.59	< 0.001
HKSA (°)	4.89 ± 1.62	5.33 ± 1.79	− 0.44 (− 0.75 to − 0.13)	− 0.27	0.005
LDFA (°)	92.43 ± 2.33	92.31 ± 2.30	0.11 (− 0.17–0.40)	0.08	0.431
MPTA (°)	84.92 ± 3.87	84.60 ± 3.60	0.31 (− 0.22–0.84)	0.11	0.246
Femoral Torsion (°)	14.14 ± 7.04	14.81 ± 7.23	− 0.67 (− 1.78–0.44)	− 0.12	0.232
Tibial Torsion (°)	37.95 ± 3.28	35.19 ± 4.51	2.76 (1.80–3.73)	0.55	< 0.001

3D, three-dimensional; CI, confidence interval; mFTA, mechanical femorotibial angle; FEA, flexion–extension angle; FTR, femorotibial rotation; HKSA, hip-knee-shaft angle; LDFA, lateral distal femoral angle; MPTA, medial proximal tibial angle.

In the analysis of inter-observer reliability, all values except FTR and tibial torsion demonstrated good or very good reliability. In the anterior position, FTR demonstrated moderate reliability (ICC, 0.504; 95% CI, 0.271–0.662) and tibial torsion demonstrated poor reliability (ICC, 0.187; 95% CI, -0.194 to 0.447). In the posterior position, both FTR (ICC, 0.335; 95% CI, 0.023–0.547) and tibial torsion (ICC, 0.357; 95% CI, 0.055–0.562) demonstrated poor reliability (Table 2).

**Table 2:** Inter-observer correlation (95% CI) of the alignment parameters on the EOS 3D system according to the posture of indexed lower extremity.

	Inter-observer correlations (95% CI)	
	Anterior	Posterior
mFTA (°)	0.740 (0.617–0.823)	0.720 (0.589–0.809)
FEA (°)	0.984 (0.977–0.989)	0.982 (0.974–0.988)
FTR (°)	0.504 (0.271–0.662)	0.335 (0.023–0.547)
HKSA (°)	0.750 (0.633–0.830)	0.897 (0.848–0.930)
LDFA (°)	0.840 (0.765–0.891)	0.854 (0.785–0.900)
MPTA (°)	0.767 (0.657–0.841)	0.867 (0.804–0.909)
Femoral Torsion (°)	0.711 (0.575–0.803)	0.769 (0.660–0.843)
Tibial Torsion(°)	0.187 (− 0.194–0.447)	0.357 (0.055–0.562)

3D three-dimensional, *CI* confidence interval, *mTFA* mechanical femorotibial angle, *FEA* flexion–extension angle, *FTR* femorotibial rotation, *HKSA* hip-knee-shaft angle, *LDFA* lateral distal femoral angle, *MPTA* medial proximal tibial angle.

In the reliability analysis between the EOS 3D and CT 3D images, only the intra-segmental parameters were compared. All measurements relating to the femur demonstrated very good reliability but those relating to the tibia did not. In the comparisons of the values in the anterior position and CT, MPTA demonstrated good reliability (ICC, 0.616; 95% CI, 0.193–0.817) and tibial torsion demonstrated fair reliability (ICC, 0.396; 95% CI, -0.269–0.712). In the comparisons between the measured values in the posterior position and CT, MPTA demonstrated good reliability (ICC, 0.704; 95% CI, 0.379–0.859) and tibial torsion had moderate reliability (ICC, 0.537; 95% CI, 0.026–0.779) (Table 3).

**Table 3:** Inter-modality correlation (95% CI) of the alignment parameters between full-length lower extremity CT 3D images and EOS 3D images.

	Inter-method correlations (95% CI)	
	CT 3D image vs EOS-anterior	CT 3D image vs EOS-posterior
HKSA (°)	0.875 (0.737–0.940)	0.906 (0.803–0.955)
LDFA (°)	0.857 (0.701–0.932)	0.836 (0.656–0.922)
MPTA (°)	0.616 (0.193–0.817)	0.704 (0.379–0.859)
Femoral Torsion (°)	0.857 (0.699–0.932)	0.819 (0.619–0.914)
Tibial Torsion (°)	0.396 (− 0.269–0.712)	0.537 (0.026–0.779)

3D three-dimensional, *CT* computed tomography, *CI* confidence interval, *HKSA* hip-knee-shaft angle, *LDFA* lateral distal femoral angle, *MPTA* medial proximal tibial angle.

**Figure 4:**
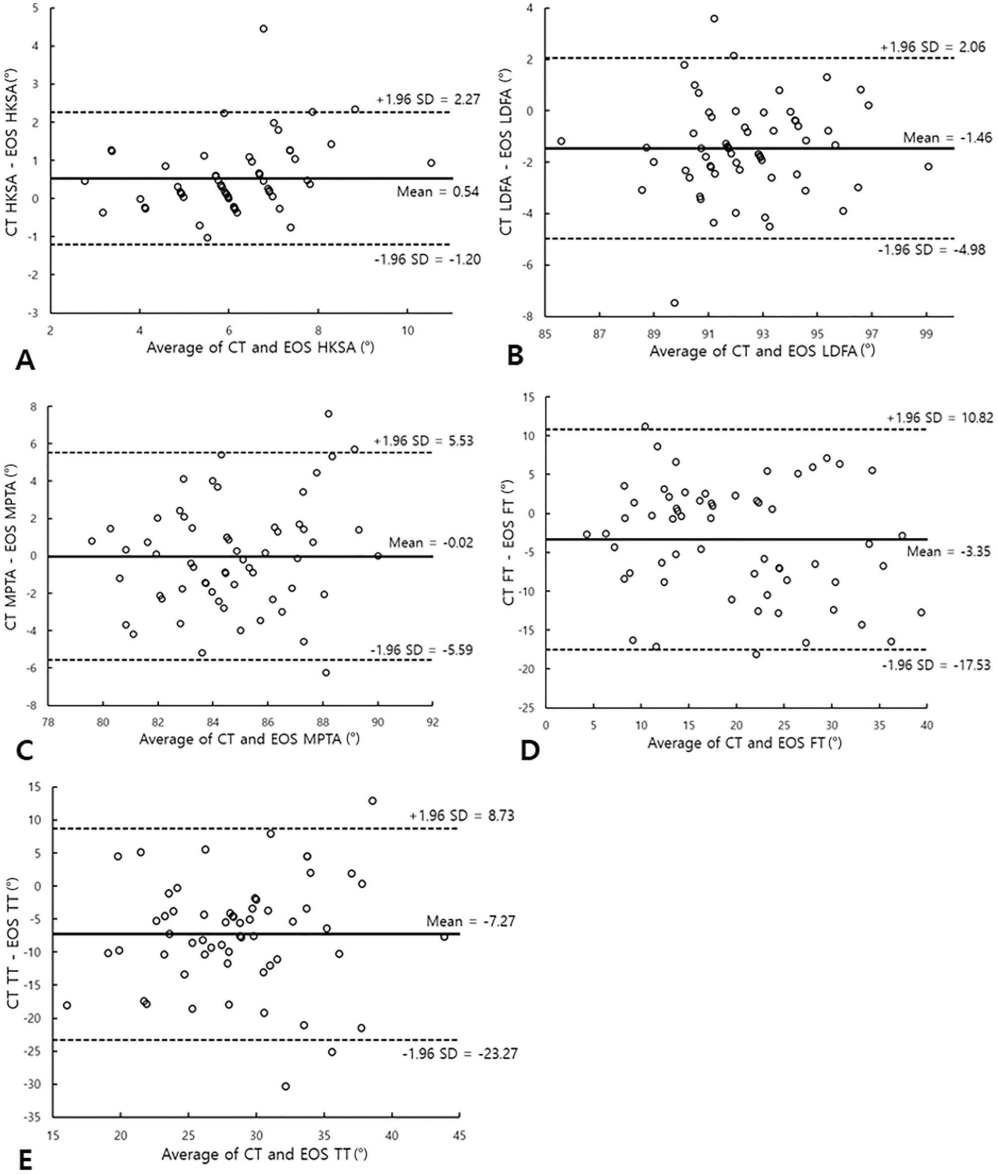
Bland–Altman plots for CT and EOS depicting the agreement between them in the assessment of the (**A**) hip-knee-shaft angle (HKSA), (**B**) lateral distal femoral angle (LDFA), (**C**) medial proximal tibial angle (MPTA), (**D**) femoral torsion (FT), and (**E**) tibial torsion (TT).

On the Bland–Altman plots, there was no tendency between the average value and the difference value in the parameters except HKSA. The mean difference (CT-EOS) was 0.54 (95% LOA: − 1.20 to 2.27) in HKS, 0.53 (95% LOA: − 4.98 to 2.06) in LDFA, − 0.02 (95% LOA: − 5.59 to 5.53) in MPTA, − 3.35 (95% LOA: − 17.53 to 10.82) in femoral torsion, and − 7.27 (95% LOA: − 23.27 to 8.73) in tibial torsion. LOA outliers included less than three cases in all parameters (Fig. 4).

## Discussion

The most important finding in the present study is that EOS imaging in a slightly rotated standing posture with an even weight-bearing on both feet demonstrated high reproducibility and reliability in all measurements except the rotational alignment of the tibia. However, in evaluating the tibial rotation, the reliability was lower than that reported in previous studies that also evaluated the reliability of the EOS system.

For accurate 3D modeling of the EOS system, the bony landmarks of each lower extremity must be clearly identified. However, accurate 3D reconstruction is impossible in the true lateral view of the standard standing posture because the bony landmarks of both lower extremities are superimposed. Therefore, conventionally, as suggested by Chaibi et al., spreading the feet slightly forward and backward was chosen to avoid superimposing the lower extremities^22^. However, since this posture does not apply an even weight over both extremities, the positional relationship between the femur and tibia may vary from that in the actual standing posture. According to Shetty et al., knee flexion deformity > 10° had a significant effect on the measurement of the coronal mechanical alignment^33^. Additionally, since the lower extremity alignment varies according to the weight-bearing position^23,24,34^, the measurement value may change each time because it is difficult to evenly bear weight for each measurement in the conventional posture. Therefore, the present study hypothesized that the most physiologic inter-segmental alignment could be obtained from the EOS images in a posture with even weight-bearing on both the lower extremities. This approach demonstrated high reliability except for parameters of tibial rotation. Furthermore, except for parameters of tibial rotation (FRT and tibial torsion), neither intra-segmental nor inter-segmental alignments demonstrated statistical differences between the indexed extremities. Intra-observer correlation also demonstrated good or very good reliability for both intra-segmental and inter-segmental alignment except for the parameters of tibial rotation. Inter-modality reliability between the EOS and CT images demonstrated good or very good reliability except for parameters of tibial torsion. Therefore, the modified posture can be an alternative method in EOS assessments, except for tibial rotational alignment, because it has the advantage of reflecting the patient’s physiologic state better than the conventional posture.

The conventional posture of spreading the feet not only causes changes in the inter-segmental alignment but also appears to affect the EOS measurements. In a study that evaluated the proximal femur and pelvis in children using the EOS system, Szuper et al. reported that the patient’s posture and position affected the ability to evaluate the torsional profile^35^. Folinais et al. measured the inter-observer error of rotational alignment of the lower extremities using the EOS system in unipodal and bipodal stances and compared them with those obtained using CT^27^. In this study, the bipodal stance refers to a posture in which both feet are placed one behind the other in the conventional manner. In the case of femoral torsion, there was no difference between the unipodal and bipodal stances (2.7° ± 4.5°, both); however, in the case of tibial torsion, the measured values in the bipodal stance (4.1° ± 3.5°) were different from those in the unipodal stance (2.9° ± 2.3°) and CT (2.7° ± 5.4°). In other words, since the tibial torsion value may differ from the actual value on CT in the conventional posture where the weight cannot be evenly loaded, it should be recognized that there may be differences depending on the posture when measuring with the EOS system using the conventional method. In our study, a physiological, even, weight-bearing posture was used; however, it did not demonstrate good reliability in relation to tibial torsion.

As mentioned above, rotational alignments ​​of the tibia demonstrated inferior reproducibility and reliability compared to other measurements. FTR and tibial torsion demonstrated statistical differences according to the position of the indexed extremity, and inter-observer correlations revealed low ICC values ​for FRT and tibial torsion. In comparison with the CT 3D model, the ICC of tibial torsion demonstrated the lowest value, and the mean difference was also the largest (-7.27°). These results are consistent with those in the existing literature. Guenoun et al. evaluated the reliability of the EOS system in 25 patients with total hip arthroplasty and reported that the inter-observer and intra-observer correlations were higher in femoral rotation (anteversion, 0.821 and 0.912) than in FTR (0.652 and 0.719) and tibial torsion (0.730 and 0.826)^36^. In a study by Buck et al. in which they compared the EOS system and CT in 35 patients, tibial torsion was greater than femoral torsion in the average difference between readers (0.8° vs 0.1° in EOS) and the average difference between CT and EOS images (3° vs 0°)^37^. In a study by Folinais et al. that included 43 lower limbs of 30 people, inter-observer reproducibility of the EOS system was lower in the tibia than in the femur (0.86 vs 0.93), and the inter-observer error was also larger in the tibia than in the femur (3.4° vs 2.7°)^27^. These studies concluded that the EOS imaging system is equivalent to CT and has high reliability in measuring the rotational alignment of the lower extremities; however, tibial rotation is less accurate than femoral rotation. This is due to the anatomical structure of the tibia and the measurement method in the EOS system. Since the EOS system’s 3D reconstruction is semi-automatically performed by adjusting the standard bone segment provided by the software to fit the patient's anatomical structure, it increases the risk of errors^38^. The fibula and tibia are referenced as the anatomical structures when reconstructing the 3D model in the EOS system, which require more adjustments of the standard bone segments than those in the femur; therefore, the segments overlap and it is difficult to distinguish them. Additionally, since the anatomy of the tibia includes various variations on the plateau^39^, it is difficult to accurately control this segment, which results in a large error between the measurements. Particularly, in the rotated posture used in our study, the statistical model based on normal bones cannot be used accurately; therefore, the agreement between test modalities was lower^40^.

Our study has several limitations. First, this was a retrospective study, which may be prone to the risk of selection bias. Second, since the number of patients was small, the variability between them may be higher, which may have affected the reliability. Third, semi-automated 3D images of the EOS system rely on the manual identification of anatomical landmarks; therefore, this system is prone to more errors than with 3D CT reconstructions. Nevertheless, this study has the advantage that it was the first to evaluate the EOS system in a physiologic posture with an even load-bearing on both lower extremities, unlike the preexisting method. The results of this study are significant because they suggest a new method to evaluate more accurately some of the parameters of lower extremity alignment using the EOS system.

## Conclusions

For the coronal and sagittal alignment parameters measured by the EOS 3D system with rotated standing posture, there were no significant difference for either position of the indexed extremities with high agreement between the observers as well as with the CT 3D model. However, the measurements of FTR and tibial torsion demonstrated inconsistencies between the position of the indexed extremities, low agreement between the observers, and low agreement with the CT 3D model. Therefore, clinicians should interpret these measurements carefully.

## Data Availability

The datasets generated during and/or analysed during the current study are available from the corresponding author on reasonable request.
